# Medical Properties of the May Apple

**Published:** 1857-06

**Authors:** W. H. Letherman


### MEDICINAL PROPERTIES OF THE MAY APPLE.

REPORTED BY W. H. LETHERMAM, M. D.

WEST: CLIN: INFIRMARY,
PHILADELPHIA, Jan. 22d, 1857.

*My Dear Friend*

Having had the pleasure of reading several numbers of your very interesting journal, from the "far West," permit me to give you the result of my experience in a class of remedies falsely called "Eclectic," which I have thus far seen unnoticed by our fraternity.

The remedy, I at present ask your calm consideration of, is the active principle of the May Apple, called Podophyllin. I have tried and experimented during the last six months upon 400 patients, and always with the same result, as to its utility and beneficial action on the economy, in which, hitherto, the hydr. chlor. mit. was generally employed. It has advantages over the mercurial—1st. You are in no fear of producing ptyalism. 2d. If a scrofulous cachexy lurk in the system, we need not fear the developing of that loathsome disease which at present is not sufficiently considered by the profession. 3d. In the great majority of cases where calomel is called for, (except where the venereal poison has infected the system,) I vastly prefer the Podophyllin. It unlocks the hepatic secretion, not by its irritating presence, but by absorption, a direct action. I consider it especially worthy the attention of those practicing in rural districts where they are unable to see their patient but once every three or four days. As a rule, employ it where you have been in the habit of exhibiting the hydr. chlor. mit. Before long, the Spring, with its hepatic engorgements will be with us, when an ample opportunity will be afforded.

I remain, as ever, yours respectfully,

WILLIE H. LETHERMAN, Resident Physician,
West: Clin: Infirmary, Philadelphia.

CASE.

Case 1. Admitted Aug. 10, '56. James D—age 25, born in America, resides in Philadelphia, occupation, brick-maker. History—Has always been a healthy man. Went South about 18 months ago; had an attack of intermittent fever from which he never fully recovered. Then intermittent fever for 8 months. (quotidian,) made a perfect wreck of him. Symptoms—Headache, great pain (sharp) on pressure over the liver, dull and heavy pain at all times, spleen enormously enlarged, urine high colored and scanty, acidity of stomach, general debility and prostration. Treatment—Six wet cups over the region of liver and placed in bed, and the following given:

(No. 1.)

R Podophyllin, gr ij.
Potass. Carbonas. gr j.

Divide in chart No. IV. Sig: One morning and evening.

Podiphyllin does not act upon the liver when the secretions of stomach are very acid, hence combine the alkalies. Aug. 11—Bowels open very freely during the night. Placed five wet cups on the seat of tenderness, and his bowels being too much moved, I placed him upon

(No .2.)

R Podophyllin, gr ij.
Pulv. Opii. gr j.
Saach. Alba. gr x.

Divide in chart No. IV. Sig: One three times a day.

During day, mind much disturbed, took his shirt off.

Aug. 14—Much improved. I ordered twenty leeches over the liver, and to promote the bleeding by warm fomentations. His bowels not being open too much and the head remaining heavy, dull ache across the frontal region, No. 2 was stopped, and

R Podophyllin, gr x.

Potass. Carb. gr ij.

Saach. Alba. gr x.

Divide in chart No. XX. S: One three times a day.

Diet, light soups and farinaceous articles.

Aug. 16—Able to be up, "feels like a new man," right side free from pain, has no headache, bowels open three times a day, very dark and fetid, liver reduced almost to its normal size. I applied over the spleen and liver, ℞ Tinc. Iodini to promote the absorbents to establish a healthy instead of the morbid action. This was applied every day for eight days. Aug. 20—Placed on ℞ Nitro Muriatic Acid; Sig: 10 drops three times a day.

On my return from Europe my colleague informed me he was discharged perfectly well, September 4th.

P. S. A few days ago I met him on Chestnut street, and he said "he had not been sick a day since."

When he applied for admission, his skin was a dirty yellow color with a greenish tinge. His complexion now is perfectly bright and clear. Yours, W. H. L.

